# Cardiac Fibroblasts Contribute to Myocardial Dysfunction in Mice with Sepsis: The Role of NLRP3 Inflammasome Activation

**DOI:** 10.1371/journal.pone.0107639

**Published:** 2014-09-12

**Authors:** Wenbo Zhang, Xuemei Xu, Raymond Kao, Tina Mele, Peter Kvietys, Claudio M. Martin, Tao Rui

**Affiliations:** 1 Departments of Medicine and Surgery, the Affiliated People's Hospital of Jiangsu University, Zhenjiang, Jiangsu Province, China; 2 Critical Illness Research, Lawson Health Research Institute, London, Ontario, Canada; 3 Critical Care Western, Department of Medicine, Schulich School of Medicine and Dentistry, University of Western Ontario, London, Ontario, Canada; 4 Department of Physiology & Biochemistry, College of Medicine, Alfaisal University, Riyadh, Saudi Arabia; 5 Department of Pathology, Schulich School of Medicine and Dentistry, University of Western Ontario, London, Ontario, Canada; Harvard Medical School, United States of America

## Abstract

Myocardial contractile dysfunction in sepsis is associated with the increased morbidity and mortality. Although the underlying mechanisms of the cardiac depression have not been fully elucidated, an exaggerated inflammatory response is believed to be responsible. Nucleotide-binding oligomerization domain-like receptor containing pyrin domain 3 (NLRP3) inflammasome is an intracellular platform that is involved in the maturation and release of interleukin (IL)-1β. The aim of the present study is to evaluate whether sepsis activates NLRP3 inflammasome/caspase-1/IL-1β pathway in cardiac fibroblasts (CFs) and whether this cytokine can subsequently impact the function of cardiomyocytes (cardiac fibroblast-myocyte cross-talk). We show that treatment of CFs with lipopolysaccharide (LPS) induces upregulation of NLRP3, activation of caspase-1, as well as the maturation (activation) and release of IL-1β. In addition, the genetic (small interfering ribonucleic acid [siRNA]) and pharmacological (glyburide) inhibition of the NLRP3 inflammasome in CFs can block this signaling pathway. Furthermore, the inhibition of the NLRP3 inflammasome in cardiac fibroblasts ameliorated the ability of LPS-chalenged CFs to impact cardiomyocyte function as assessed by intracellular cyclic adenosine monophosphate (cAMP) responses in cardiomyocytes. Salient features of this the NLP3 inflammasome/ caspase-1 pathway were confirmed in *in vivo* models of endotoxemia/sepsis. We found that inhibition of the NLRP3 inflammasome attenuated myocardial dysfunction in mice with LPS and increased the survival rate in mice with feces-induced peritonitis. Our results indicate that the activation of the NLRP3 inflammasome in cardiac fibroblasts is pivotal in the induction of myocardial dysfunction in sepsis.

## Introduction

Sepsis and septic shock are common entities encountered in intensive care units and they are associated with high mortality rates [4], [39]. Mortality in septic patients is primarily due to multiple organ failure (MOF) rather than from the initial infection [1]. The heart is one of the organs affected in MOF and myocardial dysfunction is associated with poor outcomes [23]. Myocardial dysfunction in septic patients has been attributed to an exaggerated inflammatory response [23]. As the most important functional cells within the heart, most experimental studies addressing the mechanisms of sepsis-induced myocardial dysfunction have focused on the cardiomyocyte. For example, pro-inflammatory cytokines released by cardiomyocytes during sepsis play an important role in the induction of decreased myocardial contractility [21]. While the cardiomyocytes make up <50% of the total cell population of the heart, cardiac fibroblasts (CFs) account for up to 60%, [13], [46]. Importantly, the two cell types are spatially intermingled in the myocardium, with virtually every cardiomyocytes bordering one or more CFs. This spatial arrangement allows for cardiac fibroblast-myocyte communication or “cross-talk”. The cross-talk between the CFs and cardiomyocytes can be mediated by paracrine signalling, direct cell–cell contact via micro tubules, and through indirect interactions via the extracellular matrix [46]. These interactions are not only important in normal cardiac functioning, but they also play roles in myocardial pathologies [13], [31], [46].

Interleukin (IL)-1β is a powerful cytokine that has been reported to be involved in sepsis-induced myocardial dysfunction [5]. Proinflammatory stimuli induce the expression of pro-IL-1β, which is an inactivated form of the cytokine. The maturation and release of the active form of IL-1β are controlled by inflammasomes, which are intracellular multi-protein platforms [17]. Activation of the inflammasomes (by PAMPs or DAMPS) triggers the maturation (activation) and secretion of IL-1β, therefore engaging in the inflammatory response [17], [25].

The nucleotide-binding oligomerization domain-like receptor containing pyrin domain 3 (NLRP3) inflammasome is one of the most well characterized inflammasomes [25], [36]. It consists of the NLRP3 scaffold, which is a nucleotide-binding oligomerization domain-like receptor with a pyrin domain 3, the ASC (PYCARD) adaptor, and procaspase-1 [17]. The NLRP3 is an exceptional sensor protein that responds to diverse physical and chemical stimuli, as well as to cell stress signals, such as reactive oxygen species (ROS), extracellular adenosine triphosphate (ATP) [9], [22]. As a common substrate of various inflammasomes, pro-caspase-1 is converted to its active form, caspase-1 (consisting of p20 and p10 heterotetramers). Caspase-1 cleaves intracellular pro-IL-1β to yield the mature, active IL-1β (p17), which is secreted into the extracellular space [34].

The NLRP3 inflammasome is predominantly expressed in CFs, while the levels of the NLRP3 inflammasome in the cardiomyocytes are limited [27]. Inappropriate activation of the NLRP3 inflammasome in CFs has been implicated in ischemia/reperfusion-induced myocardial injury [27]. In the present study, we provide evidence indicating that the NLRP3 inflammasome in CFs is activated in sepsis and increases IL-1β production. The CF-derived IL-1β impacts on cardiomyocyte (cardiac fibroblast-myocyte cross-talk) and impairs its contractile function.

## Materials and Methods

### Mice

C57BL/6 mice were obtained from Charles River Canada (St. Constant, QC, Canada) and were housed in the Vivarium Service of Victoria Research Laboratories with a 12-hr light/dark cycle and free access to rodent chow and tap water. The mice were used for *in vivo* experiments, and they served as a source for the isolation of CFs. Mouse breeding pairs were used to generate neonatal mice for the isolation of cardiomyocytes for the *in vitro* experiments. The experimental protocols were approved by the Western University Animal Care and Use Committee (protocol# 2006–111).

### Preparation of CFs and cardiomyocytes

CFs were derived from adult mice, as previously reported, with minor modifications [24], [30], [35]. In brief, heart tissue was dissected enzymatically (Collagenase II, 160 U/ml). After performing washing steps, the cell suspension was passed through a nylon filter (70 µm), and endothelial cells were removed with a magnetic beads technique [26]. Cells were subsequently placed in a humidified incubator gassed with 5% CO2 at 37°C for 1 hr. The adherent cells were mainly CFs, and the non-adherent cells (cardiomyocytes) were removed. Finally, the CFs were cultured with Dulbecco's Modified Eagle's Medium (DMEM)-F12 supplemented with 10% fetal calf serum (FCS), 20 mM L-glutamine and 100 U/ml penicillin G, and 100 µg/ml streptomycin. This method yielded a 95% purity of CFs, as identified by the positive staining of a fibroblast marker (ER-TR7) [32]. Cells of one through three passages were used for the experiments.

Neonatal cardiomyocytes were isolated and cultured, as previous described [44]. Briefly, hearts were harvested, minced, and digested with Collagenase II. After a washing step, the cells were suspended in M199 with 10% FCS. The myocytes were enriched by a pre-plating approach (to remove contaminating cells) before being seeded into cell culture plates. After 72 hrs in culture, the cells had formed a confluent monolayer consisting of 95% myocytes beating in synchrony, and they were used in the experiments at this time.

### Endotoxemia/sepsis models

To study the role of NLRP3 inflammasome in myocardial inflammation and myocardial dysfunction, a mouse model of endotoxemia was induced by the intraperitoneal (i.p.) injection of 0.5 ml of normal saline containing lipopolysaccharides (LPS, 10 mg/kg) to 3-month-old mice, as previously described [41]. As an *in vitro* correlate of the mouse model of endotoxemia, CFs were exposed to DMEM F12 with LPS (LPS, 1 µg/ml). In addition, a multimicrobial sepsis model was established using feces-induced peritonitis (FIP) approach. The FIP was induced by an injection (i.p.) of 0.5 ml of pooled fecal material (30 mg/ml in normal saline) to 3-month-old mice [43]. As this was a more severe model, pain control was managed with subcutaneous injection of buprenorphine (50 µg/Kg, every 12 hrs).

NLRP3 inflammasome blockade. Both pharmacologic and genetic blockade approaches were used to assess the role of NLRP3 inflammasome in the endotoxemia/sepsis models. Glyburide is a sulfonylurea drug used for the treatment of type 2 diabetes. It inhibits adenosine triphosphate (ATP)-sensitive K^+^ channels, and it is the first identified compound to inhibit the NLRP3 inflammasome [16]. In addition, NLRP3 silencing approach was employed using small interfering RNA (siRNA).

### Experimental protocols

Mice (16 mice per group) were randomly allocated to the following 4 groups: sham, LPS, LPS with glyburide as well as glyburide only. Glyburide (Invivogen, San Diego, CA, USA) was given (1 mg/Kg, i.p.) 30 min before the LPS challenge. Equivalent volume (2 µl, i.p.) of vehicle (dimethyl sulfoxide, DMSO) was administered to sham and LPS group. The mice were sacrificed 8 hours after LPS injection, and plasma as well as myocardial tissue was harvested for assay. Alternatively, the mice were subjected to myocardial function evaluation 24 hours after LPS injection. FIP mice were used to assess whether inhibition of NLRP3 inflammasome could afford a survival benefit. The FIP mice were randomly divided into two groups (10 mice/group), which were injected i.p. daily with either glyburide (1 mg/Kg.) or the equal volume of DMSO in 300 µl PBS, with the first dosing being 30 min prior to FIP. The mice were monitored hourly up to 72 hrs for the following 5 parameters: general appearance (abnormal posture, lethargy, depressed appetite, hypothermia, swelling, ruffled fur, piloerection), dehydration, weight loss, labored respiration, behaviour and activity (interest in surroundings, gettingt up without prompting, normal gait, steady on feet, ability to access food and water). Each parameter scored from 0 (normal) to 4 (very abnormal). Mice with a score of 10 were monitored more frequently to identify deteriorating conditions. Mice with a score of 20 were considered to be moribund and were humanely euthanized with cervical dislocation.

As an in vitro model of endotoxemia, CFs were challenged with LPS; saline (vehicle) was added to the media as a control. To identify the optimal time window for activation of NLRP3 inflammasome, the cells were treated with LPS for 4, 8, 12 and 24 hrs. Based on the results, a 24-hour period of LPS challenge was used in the following experiments. To study the role of NLRP3 inflammasome in LPS-induced IL-1β production, glyburide (50–200 µM) or equal volume of DMSO was added to the CFs 30 min prior to LPS treatment.

### Western blot

Protein levels of NLRP3, pro-caspase-1, caspase-1 p10 (activated caspase), pro-IL-1β, and mature IL-1β were assessed in either tissue or cells using Western blot [41]. Briefly, CFs were lysed or mouse myocardial tissue were homogenized in lysis buffer (10 mM Tris [pH 7.4], 150 mM NaCl, 5 mM EDTA, 1% Triton X-100, 10 mM NaF, 1 mM Na3VO4, 10 µg/ml leupeptin, 10 µg/ml aprotinin, and 20 mM PMSF). The supernatants were processed using the BCA method, and 10-50 µg total protein for each sample was separated on 10–15% sodium dodecyl sulfate (SDS)-polyacrylamide gels (PAGE) and transferred to polyvinilide fluoride (PVDF) membranes. After blocking with 3% bovine serum albumin (Wisent Inc, St-Bruno, QC, Canada), the membranes were probed with primary antibodies against mouse NLRP3 (Adipogen, San Diego, CA, USA) at 1∶500 dilution, pro-caspase-1, caspase-1 p10 (Santa Cruz, Dallas, TX, USA) at 1∶200, or pro-IL-1β, IL-1β (R and D Systems, Minneapolis, MN, USA) at 1∶500, or tubulin (abcam, Toronto, ON, Canada) at 1∶2000. After being incubated with relevant secondary antibodies, the specific bands were visualized with an enhanced chemiluminescence (ECL) detection system, and quantified with an Imaging Densitometer (Bio-Rad Laboratories, Inc., Hercules, CA, USA).

### ELISA

IL-1β in culture supernatants, mouse plasma or myocardial homogenates was detected with a BD OptEIA mouse IL-1β ELISA kit (BD Biosciences, San Diego, CA, USA) according to the manufacturer's instructions. Culture medium was centrifuged at 500 g for 5 min, and the supernatant was collected and stored at −80°C until assay. Blood samples from left common carotid artery were collected and plasma was obtained by centrifugation at 4,000 g for 10 min. Myocardial tissue from the left ventricle was homogenized in PBS containing a protease inhibitor cocktail. The homogenates were centrifuged at 10,000 g for 30 min to remove debris and insoluble material, and aliquots of the supernatants were ready for IL-1β measurements. The data are expressed at pg/mg protein, with protein concentration determined using the BCA method.

### NLRP3 siRNA

For the small interfering RNA (siRNA) assay, CFs were transiently transfected with 40 nM scrambled siRNA or NLRP3 siRNA (Life Technologies, Burlington, ON, Canada) using Lipofectamine 2000 (Life Technologies, Burlington, ON, Canada) according to the manufacturer's instructions [43]. Successful down-regulation of LPS-induced NLRP3 expression in CFs in all experiments was confirmed by Western blot analysis. 48 hrs after the procedure, the CFs with siRNA were used in the experiments involving the role of NLRP3 inflammasome in LPS-induced IL-1β production and the role of NLRP3 inflammasome in mediating CFs-cardiomyocytes interaction.

### Intracellular cyclic adenosine monophosphate (cAMP)

An increase in intracellular cAMP in cardiomyocytes/myocardial tissue in response to β-adrenergic agonist stimulation is generally associated with enhanced myocardial contractility and an impaired cAMP response to β-adrenergic agents has been reported in both *in vitro* and *in vivo* septic models [28], [29]. Thus, the role of NLRP3 in modulating the cAMP response to the β-adrenergic agonist, dobutamine in isolated cardiomyocytes was assessed. Firstly, CFs were pre-transfected with NLRP3 siRNA, or pre-treated with glyburide (200 µM), followed by a LPS challenge for 24 hrs. The various supernatants derived from LPS- or vehicle-conditioned CFs were harvested and transferred to naïve cardiomyocytes and incubated for an additional 24 hrs. The potential role of IL-1β on cAMP accumulation was studied by adding IL-1β (5 ng/mL) (Millipore, Billerica, MA, USA) to naïve cardiomyocytes as indicated. To block the effect of IL-1β, IL-1 receptor antagonist (IL-1Ra, 5 µg/ml) (Millipore, Billerica, MA, USA) was simultaneously added to the cardiomyocytes as needed. At 24 hrs post treatment, the cardiomyocytes were washed once and incubated for 15 min in physiological buffer at 37°C. Subsequently, the myocytes were treated with 7.5 µM dobutamine (Sigma-Aldrich, St. Louis, MO, USA) or vehicle for 10 minutes; after which the buffer was aspirated and replaced with 0.1 M HCl. The cell lysates were used to detect the intracellular cAMP with a cAMP direct immunoassay kit (BioVision, Milpitas, CA, USA) as per the manufacturer's instructions.

### Myocardial contractile function

24 hrs after the injection of LPS or saline, mice were anesthetized with ketamine (150 mg/kg) and xylazine (5 mg/kg), which were administered subcutaneously. A Millar tip transducer catheter (Model SPR-839, 1.4 Fr.) was advanced into the left ventricle (LV) via the right carotid artery. Pressure-volume (PV) measurements were taken during quiet respiration and recorded using a Millar PV Conductance Unit (Model MPCU-200) and Power Lab Data Acquisition System (ADInstruments). The raw pressure and volume data collected in text files by the MPCU-200 unit and Chart/Powerlab software were imported into the PVAN software (Millar Instruments, Houston, TX). LV pressure–volume loops were generated by occlusion of the inferior caval vein [11], [41]. The LV end-systolic pressure-volume relation (ESPVR) was calculated and used as an index of myocardial contractile function [41], [42].

### Statistical analysis

All data are expressed as the mean ± standard error of the mean. Statistical analysis was performed using analysis of variance and Student's *t*-test (with a Bonferroni correction for multiple comparisons), as well as with the Kaplan–Meier test.

## Results

### Challenging CFs with LPS activates NLRP3 inflammasome and results in caspase-1 activation and increased IL-1β maturation and release

**Figure 1:**
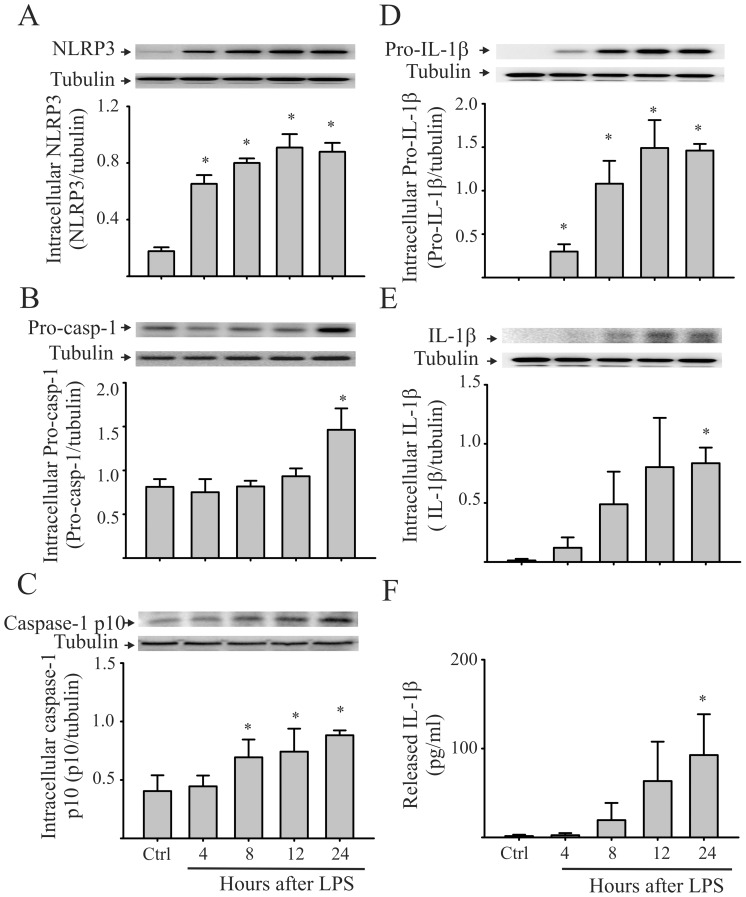
Treatment of cardiac fibroblasts (CFs) with LPS activates the NLRP3 inflammasome and results in the maturation (activation) and release of IL-1β. Mouse CFs were challenged with LPS (1 µg/ml) or saline (control). At the times indicated, the CFs were collected, lysed, and processed for the measurement (Western blot) of intracellular NLRP3 (A), pro-caspase-1 (B), activated caspase-1 p10 (C), pro-IL-1β (D), and IL-1β (E). Released IL-1β was also assessed by ELISA of the supernatants (F). For A through E, representative blots and densitometric analyses are shown. n = 3 for all experiments (A–F), *p<0.05, compared with control.

The NLRP3 inflammasome is a molecular platform that is activated upon cellular infection or stress and triggers the maturation (activation) and secretion of IL-1β, to engage the inflammatory response [17]. In order to determine whether LPS treatment activates the NLRP3 inflammasome and subsequent processing and secretion of IL-1β in CFs, we measured the intracellular levels of NLRP3, pro-caspase-1, caspase-1 p10, pro-IL-1β, as well as the intracellular and released IL-1β. As shown in **Figure 1**, levels of intracellular NLRP3, pro-caspase-1, and pro-IL-1β following the LPS treatment of CFs were increased (**Figures 1A, 1B and 1D**). Moreover, LPS treatment resulted in caspase-1 activation, as indicated by increased intracellular caspase-1 p10 (**Figure 1C**) and IL-1β maturation and release into the extracellular milieu (**Figures 1E and 1F**). These results are consistent with the NLRP3 inflammasome/caspase-1 pathway being operative in LPS-challenged cardiac fibroblasts.

**Figure 2:**
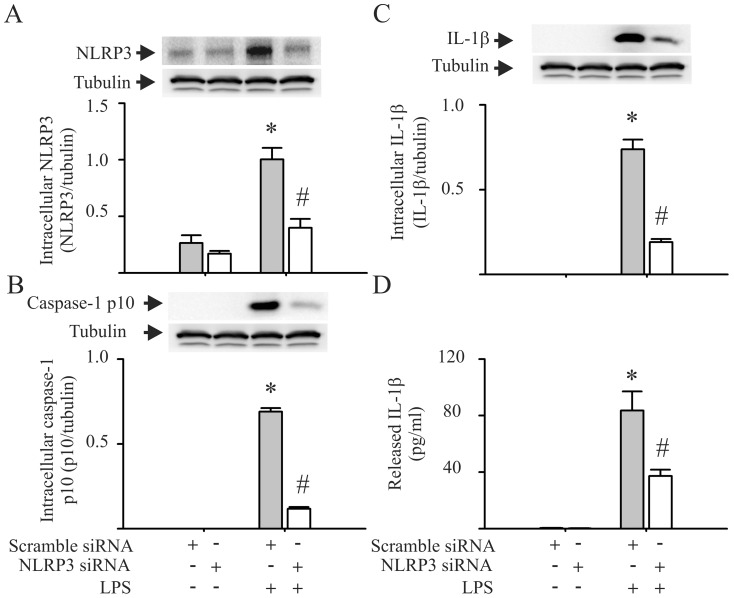
siRNA knock down of NLRP3 prevents LPS-induced caspase-1 activation and IL-1β production by cardiac fibroblasts (CFs). CFs were transfected with siRNA specific to NLRP3 or with scrambled siRNA. Forty eight hrs after transfection, the cells were challenged with LPS (1 µg/ml) or vehicle for 24 hrs. The cells were lysed for the detection of intracellular NLRP3 (**A**), caspase-1 p10 (**B**), and intracellular IL-1β (**C**) with Western blot. The supernatants were harvested for the detection of released IL-1β with ELISA (**D**). For **A**, **B**, and **C**, representative blots and densitometric analyses are shown. n = 3 for all experiments (A–D). *p<0.05 compared with control (no LPS challenge); #p<0.05 compared with the scrambled siRNA in the LPS-challenged CFs.

**Figure 3:**
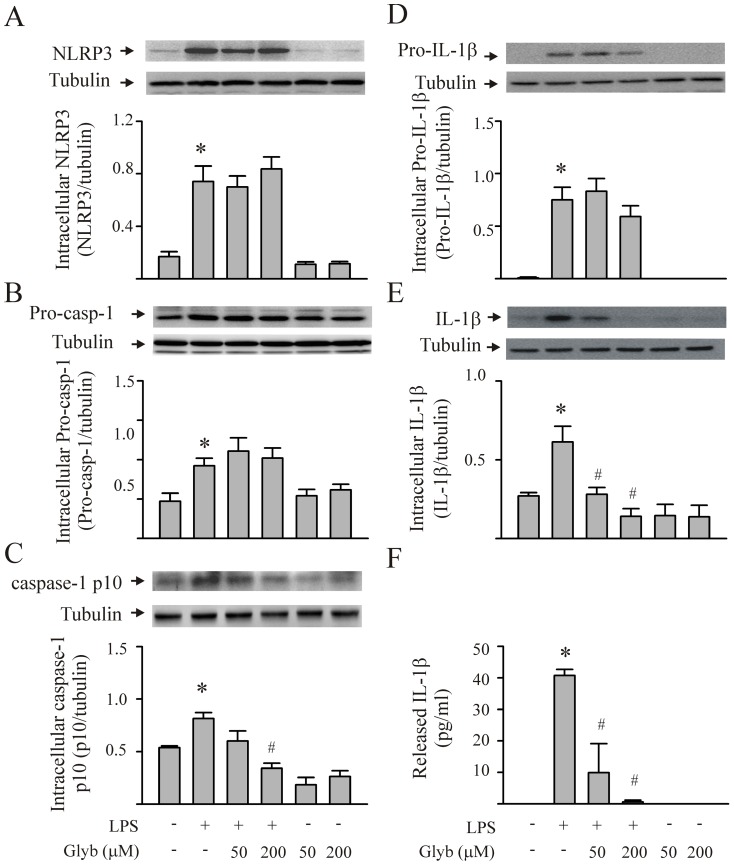
Inhibition of the NLRP3 inflammasome with glyburide (Glyb) prevents caspase-1 activation and IL-1β production by LPS-challenged cardiac fibroblasts (CFs). CFs were pretreated with either vehicle or glyburide (50 µM or 200 µM) 30 min before the LPS (1 µg/ml) challenge. Twenty-four hrs after LPS stimulation, the cells were harvested and processed for the measurement (Western blot) of intracellular NLRP3 (A), pro-caspase-1 (B), activated caspase-1 p10 (C), pro-IL-1β (D), and mature IL-1β (E). The supernatants were harvested for the detection of released IL-1β with ELISA (F). The basal levels of the various components of the NLRP3 inflammasome/IL-1β axis were not affected by glyburide. For A through E, representative blots and densitometric analyses are shown. n = 3 for all experiments (A–F). *p<0.05 compared with controls; #p<0.05 compared with LPS treatment alone.

To investigate the role of the NLRP3 inflammasome in LPS-induced IL-1β production by the CFs, the cells were transfected with siRNA, which was specific for NLRP3, or CFs were pretreated with glyburide (an inhibitor of NLRP3 inflammasomes) prior to the LPS challenge [16]. Transfection of the CFs with siRNA specific to NLPR3 blocked the LPS-induced increase in intracellular NLRP3 expression by approximately 70% (**Figure 2A**). Moreover, the NLPR3 siRNA prevented the LPS-induced activation of caspase-1 (**Figure 2B**), as well as the maturation and release of IL-1β (**Figures 2C and 2D**). Further, pretreatment of the CFs with glyburide had no effect on LPS-induced increases in the intracellular expression of NLRP3, pro-caspase-1 and pro-IL-1β (**Figures 3A, 3B and 3D**). However, glyburide prevented LPS-induced caspase-1 activation (**Figure 3C**), and it attenuated the LPS-induced IL-1β maturation and release by the CFs (**Figures 3E and 3F**) in a dose-dependent manner.

### Activation of NLRP3 inflammasome in CFs by LPS negatively regulates intracellular cAMP response to dobutamine in cardiomyocytes

**Figure 4:**
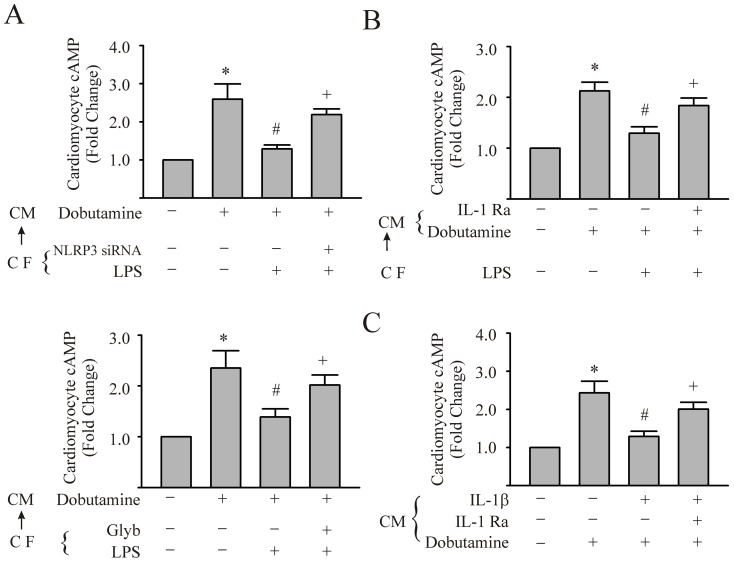
Supernatants from LPS-challenged cardiac fibroblasts (CFs) can inhibit the increase in cardiomyocyte (CM) cAMP induced by dobutamine; an effect dependent on an intact NLRP3 inflammasome/IL-1β axis in CFs. In panels A–C, CM were challenged with supernatants from LPS- or vehicle- conditioned CFs.(CF→CM). In Panel A, the CFs were transfected with NLRP3 siRNA or glyburide (200 µM) prior to challenge with LPS for 24 hrs. Subsequently, the supernatants were added to CM monolayers and thereafter, the cardiomyocytes were stimulated with vehicle or dobutamine (7.5 µM) for 10 min. The CM were harvested for the measurement of intracellular cAMP. In Panel B, CM were challenged with supernatants from LPS- or vehicle- conditioned CFs, with or without IL-1Ra (5 µg/ml) and intracellular cAMP assessed after dobutamine treatment. In panel C, CM were challenged with IL-1β (5 ng/mL) or IL-1β plus IL-1Ra (5 µg/mL) for 24 hrs. Subsequently, the CM were challenged with dobutamine and cAMP of CM was assessed. For all experiments, n = 3 and *, #, +p<0.05 compared with previous bar in the histogram.

Since the contractile activity of cardiomyocytes in our culture system was not amenable to quantitation, an indirect index of myocyte contractile function was assessed, i.e., intracellular cAMP. cAMP elevation, particularly through β-adrenergic receptor (β-AR) stimulation, has crucial positive cardiac inotropic effect. Further, the NLRP3 inflammasome is predominantly expressed in CFs, while the levels of the NLRP3 inflammasome in the cardiomyocytes are limited [27]. To determine the role of NLRP3 inflammasome activation in CFs on the function of cardiomyocytes, the cardiomyocytes were challenged with the supernatants of CFs that were conditioned with different challenges. Subsequently, the intracellular levels of the cAMP of cardiomyocytes in response to dobutamine were assessed. As shown in **Figure 4A**, cAMP levels in cardiomyocytes more than doubled after challenge with dobutamine compared with control. Supernatants of the CFs pre-conditioned with LPS blunted the dobutamine-induced elevation of intracellular cAMP by 80% (**Figure 4A**). This inhibitory effect was partially reversed (≈60% reversal) by the pretreatment of the LPS-conditioned CFs with either NLRP3 siRNA or glyburide (Figure 4A). These data indicate that the activation of the NLRP3 inflammasome in CFs negatively affects the cardiomyocyte cAMP response to dobutamine.

In another series of experiments, either IL-1Ra (5 µg/ml) was introduced to the supernatant of LPS-conditioned CFs, or IL-1β (5 ng/ml) was directly added to the culture medium of cardiomyocytes. As shown in **Figure 4B**, the negative impact of the supernatants from the LPS-conditioned CFs on the cardiomyocytes response to dobutamine was partially reversed by IL-1Ra (≈55% reversal). Moreover, IL-1β prevented the dobutamine-induced increase in intracellular cAMP in cardiomyocytes. The inhibitory effect of the IL-1β was also partially reversed by IL-1Ra (≈55% reversal) (**Figure 4C**). The results suggest that the NLRP3 inflammasome/IL-1β axis mediates the CF–cardiomyocyte interaction, and can inhibit the increase in cAMP induced by dobutamine.

### Activation of the NLRP3 inflammasome contributes to myocardial contractile dysfunction in endotoxemic mice

**Figure 5:**
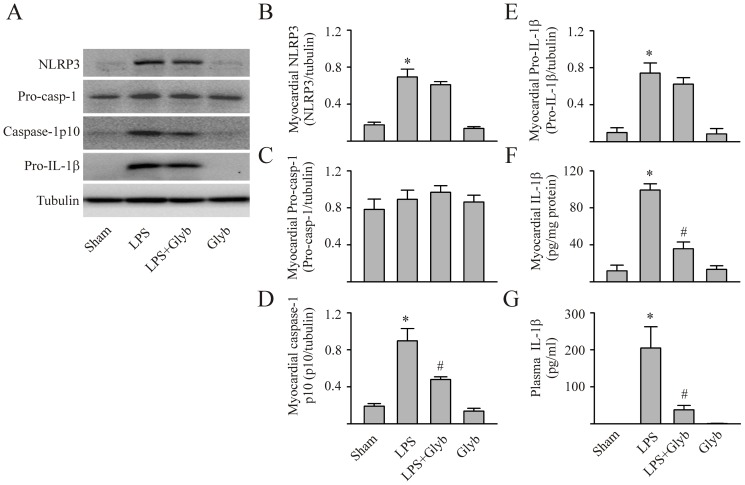
Inhibition of the NLRP3 inflammasome prevents LPS-induced increase in myocardial and circulating IL-1β in mice. Mice were injected (i.p.) with saline (sham), LPS (10 mg/kg), LPS with glyburide (1 mg/Kg), or glyburide alone. Eight hours later, myocardial tissue was harvested for measurement (Western blot) of NLRP3, pro-caspase-1, activated caspase-1p10, pro-IL-1β and IL-1β. Representative Westerns shown in panel A and densitometric analyses in panels B–F. Mature (activated) Il-1β in myocardial homogenates and plasma assessed with ELISA, panels F and G, respectively. n = 5 for all experiments; *p<0.05 compared with sham, #p<0.05 compared with LPS.

**Figure 6:**
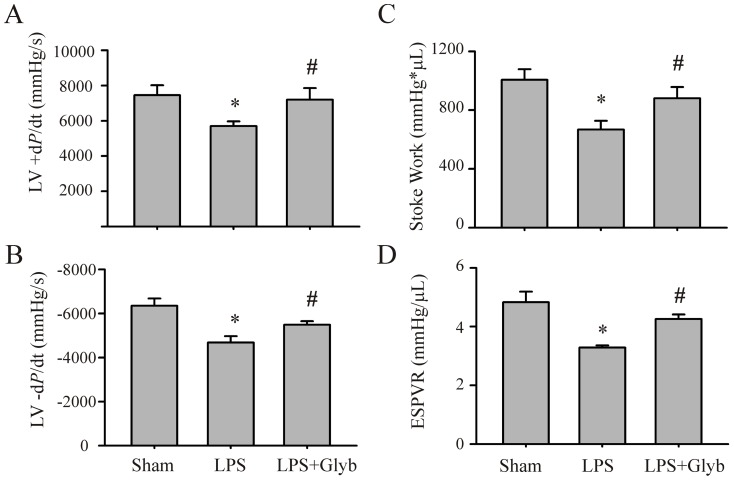
Inhibition of the NLRP3 inflammasome prevents myocardial contractile dysfunction in LPS-challenged mice. Mice were injected (i.p.) with saline (sham) or LPS (10 mg/Kg), or with LPS plus glyburide (1 mg/Kg). Myocardial contractile function (+dP/dt, -dP/dt, stroke work, and ESPVR) were assessed 24 hrs after LPS challenge with a mouse pressure–volume analysis system. N = 6 for each group; *p<0.05 compared with sham, #p<0.05 compared with LPS-challenged mice.

To determine whether studies using isolated CFs and cardiomyocytes could be translated to an *in vivo* setting, mice were either challenged with LPS (10 mg/kg) or LPS with glyburide (1 mg/kg). Mice challenged with vehicle served as the control. Myocardial NLRP3 inflammasome activation, myocardial and circulating IL-1β, and cardiac function were assessed. As shown in **Figure 5**, the levels of myocardial NLRP3 increased 8 hrs post LPS challenge in mice. Although pro-caspase-1 levels were unaltered, caspase-1 p10 levels were increased indicating that the NLRP3 inflammasome was activated. Further, LPS increased myocardial levels of activated IL-1β as well as circulating IL-1β. Glyburide prevented 1) the LPS-induced activation of the myocardial capsase-1 and IL-1β, indicating a reduction in NLRP3 inflammasome activity, as well as 2) the increase in myocardial and circulating IL-1β. Moreover, myocardial contractility was decreased in endotexemic mice (LPS-challenged), as indicated by the decrease in LV ±dP/dt, stroke work and ESPVR (**Figure 6**). Glyburide treatment attenuated the development of myocardial dysfunction in mice with LPS (**Figure 6**).

**Figure 7:**
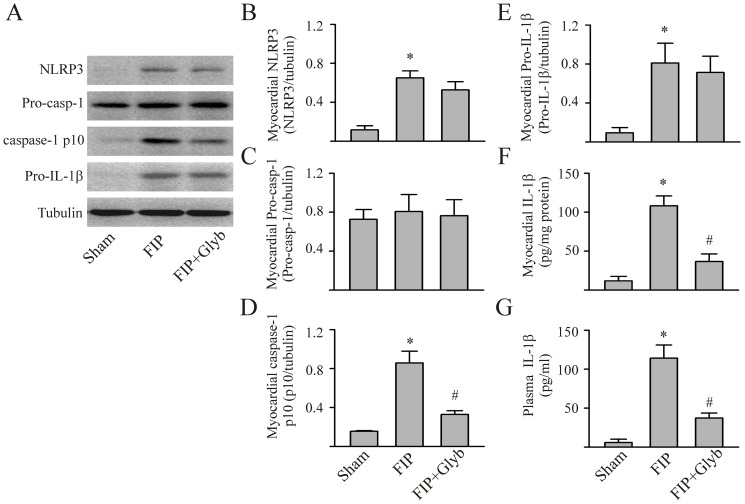
Sepsis-induced increases in myocardial and circulating IL-1β is reduced by inhibition of the NLRP3 inflammasome. Polymicrobial sepsis (FIP) was induced by i.p. injection of 0.5 ml of fecal material (30 mg/ml). Myocardial tissue was harvested for measurement (Western blot) of NLRP3, pro-caspase-1, activated caspase-1 p10, and pro-IL-1β. Representative Westerns are shown in panel A and densitometric analyses in panels B-E. Mature (activated) IL-1β in myocardial homogenates and plasma assessed with ELISA, panels F and G, respectively. n = 5 for all experiments; *p<0.05 compared with sham, #p<0.05 compared with FIP.

To assess whether the NLRP3 inflammasome/caspase-1 pathway would be activated in a polymicrobial model of sepsis we used the FIP model. In line with the LPS-induced endotoxemia model, the levels of myocardial NLRP3 and pro-IL-1β were also increased 8 hrs post-FIP induction (Figure 7). Activation of NLRP3 inflammasome was evidenced by the increased myocardial levels of caspase-1 p10 and IL-1β as well as circulating IL-1β. Glyburide prevented the FIP-induced activation of the myocardial NLRP3 inflammasome as well as FIP-induced increase in myocardial and circulating IL-1β (**Figure 7**).

**Figure 8:**
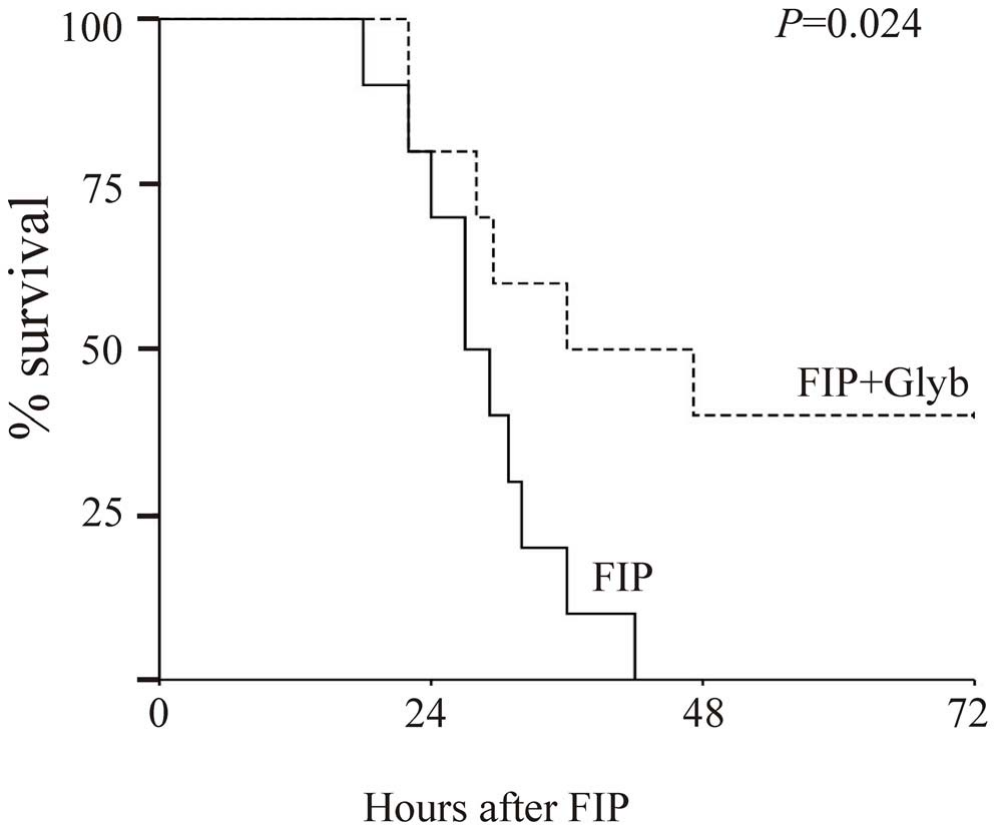
Sepsis-induced lethality is diminished by inhibition of the NLRP3 inflammasome. Polymicrobial sepsis (FIP) was induced by i.p. injection of 0.5 ml of fecal material (30 mg/ml) in 20 mice; 10 of these mice also received glyburide (1 mg/Kg, i.p. daily). The mice were monitored hourly for up to 72 hrs, and the survival rate was calculated. The survival rate of the two groups was compared with the Kaplan–Meier test, p = 0.024.

Previous studies have demonstrated that NLRP3^−/−^ mice are resistant to LPS-induced lethality [20]. In accord with the role of NLRP3 inflammasome in endotoxemia, glyburide significantly delays LPS-induced lethality in mice [16]. In order to determine whether the inhibition of the NLRP3 inflammasome has beneficial effects on a multimicrobial (peritonitis) mouse model of sepsis, we used the FIP model. As shown in **Figure 8**, all FIP mice in vehicle-treated group died within 48 hrs after induction of FIP, while FIP mice treated with glyburide daily showed a significant increase in survival rate.

## Discussion

Myocardial dysfunction frequently accompanies severe sepsis and septic shock. It is now clear that cardiac dysfunction, as evidenced by biventricular dilatation and reduced ejection fraction, is present in most patients with severe sepsis [23]. Myocardial dysfunction does not appear to be due to myocardial hypoperfusion, but rather to an exaggerated inflammatory response [23]. However, the mechanisms and signaling pathways involved in eliciting the sepsis-induced inflammatory response remain elusive. An increasing body of evidence indicates that the NLPR3 inflammasome/caspase-1/IL-1β pathway may be involved. For instance, IL-1β is increased in both human and animal models of sepsis and septic shock [10] and an IL-1 antagonist attenuates the hemodynamic and metabolic manifestations of septic shock [6], [7]. Further, genetic deletion or pharmacological inhibition of caspase-1 protects against endotoxemic shock in rodents [2], [18]. Finally, mice deficient in components of the inflammasome complex (ASC or NLPR3) are more resistant to the lethal effects of endotoxin [19], [20]. Although these isolated observations support a potential role for the NLRP3 inflammasome/caspase-1/IL-1β pathway, the present study is the first to provide a systematic assessment of this pathway in endotoxemia/sepsis both *in vivo* and *in vitro*. Further, we show that blockade of the NLRP3 inflammasome/caspase-1/IL-1β pathway can afford protection against lethality in a model of polymicrobial sepsis. Finally, we provide evidence for cardiac fibroblast-myocyte cross-talk in the development of the sepsis-induced inflammatory response.

Both IL-1β and TNF-α appear to play important roles in endoxemia/sepsis-induced myocardial dysfunction. A concentration-dependent depression of contractility induced by IL-1β or TNF-α has been reported in *in vitro* or *ex vivo* studies in human and animal myocardial tissue [3], [8], [15], [37]. The focus of the present study was on the NLRP3 inflammasome/caspase-1/IL-1β pathway. Of relevance, immunoabsorption of IL-1β can ameliorate cardiomyocyte depressant activity of human septic serum [15]. The cellular sources of IL-1β within the heart remain unclear at present. Inflammatory cells (e.g. macrophages, neutrophils) are well-documented sources of IL-1β production [45], [47]. In addition to these inflammatory cells, our data indicate that CFs are important cells within the heart that produce IL-1β. This finding is consistent with emerging evidence that CFs share many similarities with inflammatory cells in terms of how NLRP3 inflammasome/IL-1β axis is activated and functions [14], [27], [33]. Although CFs comprise two-thirds of the cell population in the heart, they have not attracted much attention except in relation to processes related to myocardial fibrosis and remodeling. In recent years, however, several studies have demonstrated CFs could act as “sentinel” cells that sense danger signals and interact with other cells such as cardiomyocytes, vascular cells, and inflammatory cells in a paracrine manner [14], [27]. Our observations provide strong support for the contention that CFs are the major cellular source of locally produced IL-1β.

Herein, we provide evidence that the IL-1β secreted by CFs can impact on cardiomyocyte function. Since a quantitative assessment of myocyte contractile activity in our *in vitro* system was not possible, we used an indirect approach, i.e. assessment of intramyocyte cAMP levels. An increase in cAMP levels is associated with the increase in myocyte contractility induced by β-adrenergic agonists and has previously been used as an index of cardiomyocyte contractility. Impaired accumulation of cAMP in cardiomyocytes/myocardial tissue has been reported in *in vitro* and *in vivo* septic models [12], [40]. In a similar vein, we show herein that IL-1β blunted the dobutamine-induced increase in intracellular cAMP in cardiomyocytes; an effect ameliorated by IL-1Ra. Cytokine-enriched supernatant from LPS-conditioned CFs also hampered cAMP accumulation in cardiomyocytes. Importantly, the role of CF NLRP3 inflammasome was highlighted because the decrease in cAMP production could be attenuated by pretreatment of the LPS-conditioned CFs with either NLRP3 siRNA or glyburide. However, it must be noted that the reversal afforded by inhibition of the NLRP3 inflammasome pathway was only partial. Thus, other, as yet unidentified, factors/agents generated by LPS (or IL-1β) are likely to be involved in attenuating the dobutamine-induced increase myocyte cAMP.

In general, the basal level of NLRP3 is not sufficient for NLRP3 inflammasome activation [17]. Therefore, most of the previous studies probing the mechanisms involved in the activation of the NLRP3 inflammasome have employed two-step approaches: a priming step (LPS treatment) followed by an activation stimulus (ATP treatment in general) [17], [27], [29]. The priming approach using LPS increases the intracellular levels of NLRP3 and pro-IL-1β. The second activation step triggers both the formation of the inflammasome complex and the activation of caspase-1. Interestingly, in the present study, we demonstrated that a single LPS challenge itself is sufficient in activating the NLRP3 inflammasome/caspase-1/inflammasome pathway in CFs. The precise explanation for the discordant observations is not readily apparent. One potential explanation is that the treatment of the CFs in the present study resulted in the simultaneous increase in NLPR3 and its activation signals, such as ROS or DAMPs.

Although our findings strongly support a role for the NLRP3 inflammasome/caspase-1/ IL-1β pathway of CFs in the myocardial dysfunction, as well as, lethality in sepsis, extrapolation to the human condition is rather tenuous. Aside from the limitations of animal and cell models, i.e., humans are more sensitive to LPS than rodents [38], myocytes in culture may not represent the situation *in situ* [14], etc., a role for IL-1β in the clinical setting is unclear at present. Clinical trials using IL-1-based therapies have been disappointing with no clear or consistent benefit in sepsis demonstrated [6], [7]. It has been suggested that grouping patients with heterogeneous conditions under the same diagnosis of “severe sepsis” contributed difficulty in reproduce results from pre-clinical experiments in clinical trials [28]. Further, the issue of synergy among various cytokines present in the “cytokine storm” of sepsis may cloud the issue. Cytokine synergy has been posited in sepsis-induced myocardial depression in previous studies [3], [15]. The combination of IL-1β and TNF-α may cause cardiomyocyte depression at concentrations 50–100 times lower than would be required if applied individually [3], [15]. In short, caution must be used in extrapolating data from pre-clinical experimental studies in cells and animals to the situation present in septic patients in the ICU. None the less, our observations suggest that the NLRP3 inflammasome may be a potential therapeutic target for the treatment of sepsis-induced myocardial dysfunction.
